# Variational Beta Process Hidden Markov Models with Shared Hidden States for Trajectory Recognition

**DOI:** 10.3390/e23101290

**Published:** 2021-09-30

**Authors:** Jing Zhao, Yi Zhang, Shiliang Sun, Haiwei Dai

**Affiliations:** School of Computer Science and Technology, East China Normal University, Shanghai 200062, China; jzhao@cs.ecnu.edu.cn (J.Z.); 51194506051@stu.ecnu.edu.cn (Y.Z.); 51131201017@stu.ecnu.edu.cn (H.D.)

**Keywords:** hidden Markov models, variational inference, trajectory recognition, Beta process

## Abstract

Hidden Markov model (HMM) is a vital model for trajectory recognition. As the number of hidden states in HMM is important and hard to be determined, many nonparametric methods like hierarchical Dirichlet process HMMs and Beta process HMMs (BP-HMMs) have been proposed to determine it automatically. Among these methods, the sampled BP-HMM models the shared information among different classes, which has been proved to be effective in several trajectory recognition scenes. However, the existing BP-HMM maintains a state transition probability matrix for each trajectory, which is inconvenient for classification. Furthermore, the approximate inference of the BP-HMM is based on sampling methods, which usually takes a long time to converge. To develop an efficient nonparametric sequential model that can capture cross-class shared information for trajectory recognition, we propose a novel variational BP-HMM model, in which the hidden states can be shared among different classes and each class chooses its own hidden states and maintains a unified transition probability matrix. In addition, we derive a variational inference method for the proposed model, which is more efficient than sampling-based methods. Experimental results on a synthetic dataset and two real-world datasets show that compared with the sampled BP-HMM and other related models, the variational BP-HMM has better performance in trajectory recognition.

## 1. Introduction

Trajectory recognition is important and meaningful in many practical applications, such as human activities recognition [1], speech recognition [2], handwritten character recognition [3] and navigation task with mobile robot [4]. In most practical applications, the trajectory is affected by the hidden features corresponding to each point. The hidden Markov model (HMM) [2], hierarchical conditional random field (HCRF) [5,6] and the HMM-based models, such as the hierarchical Dirichlet process hidden Markov model (HDP-HMM) [7], the Beta process hidden Markov model (BP-HMM) [8,9,10] and the Gaussian mixture model hidden Markov model (GMM-HMM) [2] are used to model sequential data and identify their classes [11,12,13,14].

The HMM is a popular model which has been applied widely in human activity recognition [1,15], speech recognition [2,16] and remote target tracking [2,17]. Besides, the HMM is becoming a more significant part as a building block of smart cities and Industry 4.0 [18,19] and implemented in extensive applications such as driving behaviors prediction [20] and the inernet of thing (IoT) signature anomalies [21]. One drawback of the HMM is having to ensure in advance the number of hidden states that need to be selected or cross-validated. To address this problem, several methods based on model selection are employed, such as BIC [22] or some Bayesian non-parameter prior like the BP [23] and the HDP [24]. Besides, directly using the original HMM for classification has another disadvantage, in which each HMM is trained for one class separately and thus information from different classes cannot be shared. It is worth mentioning that the sampled BP-HMM proposed by Fox et al. [9] can not only learn the number of hidden features automatically but also obtain the sharing features between different classes, which has been proved to be meaningful for human activity trajectory recognition. The sampled BP-HMM learns the shared states among different classes by jointly modeling all trajectories together, in which a hidden state indicator for one trajectory with a BP prior is introduced and thus a state transition matrix for each trajectory is maintained. When used for classification, the sampled BP-HMM calculates the class-specific transition matrix by averaging the transition matrices of the trajectories from the corresponding class. However, from the perspective of performance or efficiency, if the sampled BP-HMM [1,7] is used for classification, there is still a lot of room for improvement.

From the perspective of performance, the classification procedure in the sampled BP-HMM [1] is too rough to make full use of the trained model, in which the state transition matrix for each class is calculated by averaging the transition matrixes of all the trajectories. Obviously, this will lead to the loss of information, especially when the training set has some ambiguous trajectories. For instance, a “running” class has some “jogging” trajectories. One naive method to solve it is to select the *K* best HMMs for each class. However, it will cost plenty of time to select representatives for each class. In order to take account of both performance and efficiency, we change the way of modeling data in BP-HMMs. Differently from those versions of BP-HMMs [1,8,9,10,25], in variational BP-HMMs, an HMM is created for each class instead of for each trajectory.

From the perspective of efficiency, the existing approximate inference for the BP-HMM is based on sampling methods [1,9] which often converge slowly. This drawback of the sampled BP-HMM [1] is inconvenient to practical applications. To provide a faster convergence rate than sampling methods, we develop variational inference for the BP-HMM. If the variational lower bound is unchanged or almost unchanged, the iteration will stop. To be amenable to the variational method, we use the stick-breaking construction of the BP [26] instead of the Indian buffet process (IBP) construction [27] in the sampled BP-HMM.

In this paper, we propose a variational BP-HMM for trajectory recognition, in which the way of the data modeling and the inference method are novel compared with the previous sampled BP-HMM. On the one hand, the new method of modeling trajectories enables the model to obtain better classification performance. Specifically, the hidden state can be optionally shared, and the class-specific state indicator is more suitable for classification than the trajectory-specific state indicator in the sampled BP-HMM. The transition matrix is actually learned from the data instead of averaging all the trajectory-specific transitions. On the other hand, the derived variational inference of the BP-HMM makes the model more efficient. In particular, we use the two-parameter BP as the prior of the class-specific state indicator, which is more flexible than the one-parameter Indian buffet process in the sampled BP-HMM. We apply our model to the navigation task of mobile robots and human activity trajectory recognition. Experimental results on the synthetic and real-world data show that the proposed variational BP-HMM with sharing hidden states has advantages to trajectory recognition.

The remainder of this paper is organized as follows. Section 2 gives an overview of the BP and HMM. In Section 3, we review the model assumption of the sampled BP-HMM. In Section 4, we present the proposed variational BP-HMM including the model setting and its variational inference procedure. Experimental results on both synthetic and real-world datasets are reported in Section 5. Finally, Section 6 gives the conclusion and future research directions.

## 2. Preliminary Knowledge

In order to explain the variational BP-HMM more clearly, the key related backgrounds including BP and HMM will be introduced in the following sub-sections.

### 2.1. Beta Process

The BP is defined by Hjort [28] for applications in survival analysis. It is a significant application as a non-parametric prior for latent factor models [23,26], and used as a non-parameter prior for selecting the hidden state set of the HMM [8,9,25]. At the beginning, the BP is defined on the positive real line $\left(R^{+}\right)$ then extended to more general spaces Ω (e.g., R).

A BP, $B∼\mathrm{BP}\left(\alpha ,B_{0}\right)$, is a positive Lévy process. Here, α is the concentration parameter and $B_{0}$ is a fixed measure on Ω. Let $\gamma =B_{0}\left(\Omega \right)$. The $\mathrm{BP}\left(\alpha ,B_{0}\right)$ is formulated as

(1) $$\begin{array}{c} \begin{array}{cc} & B_{K}=\sum_{k=1}^{\infty }\pi_{k}\delta_{\omega_{k}},\\ & \omega_{i_{j}}\overset{i.i.d.}{∼}\frac{1}{\gamma }B_{0}, \end{array} \end{array}$$

where {ω} are atoms in *B*. If $B_{0}$ is continuous, the Lévy measure of the BP is expressed as

(2) $$\begin{array}{c} \begin{array}{cc} & \nu \left(d\omega ,d\pi \right)=\alpha \left(\omega \right)\pi^{-1}{\left(1-\pi \right)}^{c\left(\omega \right)-1}d\pi B_{0}\left(d\omega \right). \end{array} \end{array}$$

If $B_{0}$ is discrete, in the form of $B_{0}=\sum_{k}q_{k}\omega_{k}$, the atoms in *B* and $B_{0}$ have the same location. It can be represented as follows

(3) $$\begin{array}{c} \begin{array}{cc} & B_{K}=\sum_{k=1}^{K}\pi_{k}\delta_{\omega_{k}},\\ & \pi_{k}\overset{i.i.d.}{∼}\mathrm{Beta}\left(\frac{\alpha \gamma }{K},\alpha \left(1-\frac{\gamma }{K}\right)\right),\\ & \omega_{k}\overset{i.i.d.}{∼}\frac{1}{\gamma }B_{0}. \end{array} \end{array}$$

As K→∞ and $H_{K}\to \infty$, *B* represents a BP [29].

The BP is conjugate to a class of Bernoulli process, denoted by $\mathrm{BeP}\left(B\right)$. For example, we define a Bernoulli process $F∼\mathrm{BeP}\left(B\right)$. In this article, we focus on the discrete Bernoulli process in the form of $B=\sum_{k}\pi_{k}\delta_{\omega_{k}}$, and then the Bernoulli process can be expressed as $F=\sum_{k}b_{k}\delta_{\omega_{k}}$, where B∈[0,1], $b_{k}$ is the independent Bernoulli variable with the probability $\pi_{k}$. If *B* is a BP, then

(4) $$\begin{array}{c} \begin{array}{cc} & B∼\mathrm{BP}\left(\alpha ,B_{0}\right),\\ & F∼\mathrm{BeP}\left(B\right), \end{array} \end{array}$$

is called the Beta-Bernoulli process.

Similarly to Dirichlet process which has two principle methods for drawing samples, (1) the Chinese restaurant process [30], (2) the stick-breaking process [31], the BP generates samples using the Indian buffet process (IBP) [23] and the stick-breaking process [29].

The original IBP can be seen as a special case of the general BP, i.e., an IBP is a one-parameter BP. Similarly to the Chinese restaurant process, the IBP is described in the view of customers choosing dishes. It is also employed to construct two-parameter BPs but with some details changed. Specifically, the procedure for constructing BP$\left(\alpha ,B_{0}\right),\gamma =B_{0}\left(\Omega \right)$ is as follows:

1. The first customer takes the first $\mathrm{Poisson}\left(\gamma \right)$ dishes.
2. The *n*th customer then takes dishes that have been previously sampled with probability $\frac{m_{k}}{\alpha +n-1}$, where $m_{k}$ is the number of people who have already sampled the dish *k*. He also takes Poisson $\left(\frac{\alpha \gamma }{\alpha +n-1}\right)$ new dishes.

The BP has been shown as a de Finetti mixing distribution underlying the Indian buffet process, and an algorithm has been presented to generate the BP [23].

The stick-breaking process of the BP, $B∼\mathrm{BP}\left(\alpha ,B_{0}\right)$, is provided by Paisley et al. [29]. It is formulated as follows.

(5) $$\begin{array}{c} \begin{array}{cc} & B=\sum_{i=1}^{\infty }\sum_{j=1}^{C_{i}}V_{i_{j}}^{\left(i\right)}\prod_{l}^{i-1}\left(1-V_{i_{j}}^{\left(l\right)}\right)\delta_{\omega_{i_{j}}},\\ & C_{i}\overset{i.i.d.}{∼}\mathrm{Poisson}\left(\gamma \right),\\ & V_{i_{j}}^{\left(l\right)}\overset{i.i.d.}{∼}\mathrm{Beta}\left(1,\alpha \right),\\ & \omega_{i_{j}}\overset{i.i.d.}{∼}\frac{1}{\gamma }B_{0}. \end{array} \end{array}$$

It is clearly shown from the above equations that in every round (indexed by *i*), $C_{i}$ atoms have been selected, the weights of them follow an *i*-times stick-breaking process in which each breaking has the $\mathrm{Beta}\left(1,a\right)$ probability and $C_{i}$ is drawn from $\mathrm{Poisson}\left(\gamma \right)$.

### 2.2. Hidden Markov Models

The HMM [2] is a state space model where each sequence uses a Markov chain of discrete latent variables, with each observation conditioned on the state of the corresponding latent variables. Obviously, they are appropriate to model the data varying over time, and the data can be considered to be generated by the process that switches between different phases or states at different time-points. The HMM has been proved as a valuable tool in human activity recognition, speech recognition and many other popular areas [32].

**Figure 1:**
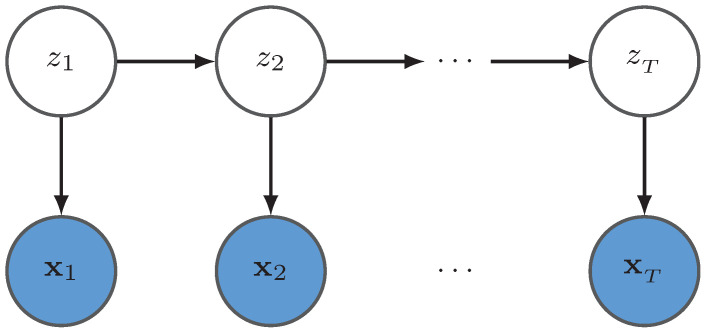
The probabilistic graphical model for an HMM where $X=\{x_{1},x_{2},\cdots ,x_{T}\}$ represents an observation sequence and $Z=\{z_{1},z_{2},\cdots ,z_{T}\}$ represents the corresponding hidden state sequence.

Suppose that the trajectory observation $X=\{x_{1},\ldots ,x_{N}\}$ is an N×d matrix and $Z=\{z_{1},\ldots ,z_{N}\}$ is a *N* dimensional latent variable vector which has a value set $\Omega_{1}$ with size *K*. The joint distribution of *X* and *Z* is expressed as

(6) $$\begin{array}{c} \begin{array}{cc} p\left(X,Z|\theta \right)= & p\left(z_{1}|\pi \right)\left\{\prod_{t=2}^{T}p\left(z_{t}|z_{t-1},\Pi \right)\right\}\\ & \prod_{t=1}^{T}p\left(x_{t}|z_{t},\phi \right), \end{array} \end{array}$$

where $\theta =\{\pi_{0},\pi_{k},\phi \}$, and *A* is a K×K matrix with $\pi_{jk}=p\left(z_{t+1}=k|z_{t}=j\right)$, $t=\left\{1,\ldots ,T-1\right\},i,j\in \Omega_{1}$ with $\sum_{k}\pi_{jk}=1$, and $\pi_{0k}$ is a *K* dimensional vector with $\pi_{0k}=p\left(z_{1}=k\right),k\in \Omega_{1}$ with $\sum_{k}\pi_{0k}=1$. Furthermore, in the nonparametric version of HMM, the matrix Π can be assumed to obey a Dirichlet distribution, i.e., $\pi_{j}∼\mathrm{Dir}\left(\alpha_{1},\alpha_{2},\cdots ,\alpha_{K}\right)$ where $\sum \alpha_{k}=1.$ The probabilistic graphical model is represented in Figure 1.

If $x_{t}$ is discrete with value set $\Omega_{2}$ in the size of *D*, ϕ is a K×D matrix with element $\phi_{i_{j}}=p\left(x_{t}=j|z_{t}=i\right),i\in \Omega_{1},j\in \Omega_{2}$. Π and ϕ are named respectively as the transition matrix and emission matrix. If $x_{t}$ is continuous, the emission matrix will be replaced by the emission distribution, where ϕ is often defined as a distribution like Gaussian distribution $p\left(x_{t}|z_{t}=k\right)=N\left(x_{t}|\mu_{k},\Sigma_{k}\right)$, $k\in \Omega_{1}$. In the fully Bayesian framework, $\mu_{k},\Sigma_{k}$ can be regarded as random variables with distribution like normal inverse Wishart or Gaussian with Gamma distribution.

Marginal likelihood is often used to evaluate how an HMM is fit for the trajectories. Therefore, the HMM is usually trained by maximizing the marginal likelihood over the training trajectories. Baum–Welch (BW) algorithm, as an EM method is a famous algorithm for learning parameters of HMMs. The parameters include the transition matrix, the initial state distribution and the emission matrix (distribution’s parameters). In the BW algorithm, the forward-backward algorithm is employed to calculate the marginal probability. It should be noted that since the BW algorithm can only find the local optimum, multiple initializations are usually used to obtain better solutions. Given the learned parameters, the most likely state sequence corresponding to a trajectory is required in many practical applications. Viterbi algorithm is an effective method to obtain the most probable state sequence.

HMMs are a kind of generative model; they model the distribution of all the observed data. In trajectory classification tasks, such as activity trajectory recognition, different HMMs are used to model different classes of trajectories separately. After training these HMMs, the parameters in different HMMs are used to evaluate the newly come trajectory to find the most probable class. Specifically, to model large multiple trajectories from different classes, a separate HMM is defined for each class of trajectories, where $\theta^{c}$ represents its parameters. Given the trained HMMs, the class label $y^{*}$ of a new test trajectory $x^{*}$ is determined according to

(7) $$y^{*}=\underset{c}{\arg\max}\ln p\left(x^{*}|\theta^{c}\right),$$

where $p\left(x^{*}|\theta^{c}\right)$ can be calculated using the forward-backward algorithm.

## 3. The Sampled BP-HMM

The sampled BP-HMM [9] is proposed to discover the available hidden states and the sharing patterns among different classes. It jointly models multiple trajectories and learns a state transition matrix for each trajectory. The sampled BP-HMM is successfully applied to trajectory recognition tasks, such as human activity trajectory recognition [1,10].

The sampled BP-HMM uses HMMs to model all the trajectories from all the classes and uses the BP as the prior of the indicator variables with each one corresponding to one trajectory. Suppose $X=\left\{X^{\left(1\right)},X^{\left(2\right)},\ldots ,X^{\left(n\right)},\ldots ,X^{\left(N\right)}\right\},N\in N^{+}$ where $X^{\left(n\right)}$ is the nth trajectory. Each trajectory is modeled by an HMM. These HMMs share a global hidden feature set Ω with the size of *∞*. The sampled BP-HMM uses a hidden state selection matrix *F* with the size of N×∞ to indicate the available states for each trajectory, i.e., $f_{nk}=\left\{0,1\right\}$ indicators whether the nth HMM owns the kth state. The prior of the transition matrix $\Pi^{\left(n\right)}$ for each trajectory is related to *F*, The transition matrix of the nth HMM is

(8) $$\pi_{j}^{\left(n\right)}∼Dir\left(\left[r,r,\ldots ,r+\kappa ,r,\cdots \right]⊙f_{n}\right),j>1,$$

and the initial state probability vector $\pi_{0}^{\left(n\right)}$ is also related to *F*,

(9) $$\pi_{0}^{\left(n\right)}∼Dir\left(\left[r,r,\ldots ,r,r,\cdots \right]⊙f_{n}\right).$$

Similarly to the standard HMM, the latent variable $z^{\left(n\right)}$ is a discrete sequence with

(10) $$z_{1}^{\left(n\right)}∼\pi_{0}^{\left(n\right)},z_{t+1}^{\left(n\right)}|z_{t}^{\left(n\right)}∼\pi_{z_{t}^{\left(n\right)}}^{\left(n\right)},t=1\ldots T,$$

and the emission distribution of the nth HMM is

(11) $$\begin{array}{c} \begin{array}{cc} & X_{t}^{\left(n\right)}|z_{t}^{\left(n\right)}∼N\left(\mu_{z_{t}^{n}},\Sigma_{z_{t}^{n}}\right),\\ & \left(\mu_{k},\Sigma_{k}\right)∼\mathrm{NIW}\left(u_{0},\lambda_{0},\Phi_{0},\upsilon_{0}\right). \end{array} \end{array}$$

In order to build a non-parameter model, the hidden states selection matrix *F* is constructed by a BP−BeP.

(12) $$\begin{array}{c} \begin{array}{cc} & B∼\mathrm{BP}\left(\alpha ,B_{0}\right),\\ & f_{i}|B∼\mathrm{BeP}\left(B\right). \end{array} \end{array}$$

From the perspective of the characteristic of BPs, we can find that the greater the concentration parameter α, the sparser the hidden state selection matrix *F*, and greater γ will lead to more hidden features.

Given the above model assumptions, the sampled BP-HMM uses the Gibbs sampling method to train the model and uses the gradient based method to learn the parameters. With the state transition matrix for each trajectory being learned, the average state transition matrix for each class can be calculated by the mean operation. The new test trajectories are classified according to their likelihood probabilities conditional on each class.

## 4. The Proposed Variational BP-HMM

In this section, we will introduce the proposed variational BP-HMM which has more reasonable assumptions and more efficient inference procedure than the sampled BP-HMM. We first describe key points of our model and present our stick-breaking representation for the BP which allows for variational inference. Then we give the joint distribution of the proposed BP-HMM and the variational inference for the BP-HMM.

### 4.1. BP-HMM with the Shared Hidden State Space and Class Specific Indicators

As introduced above, the existing sampled BP-HMM can jointly learn the trajectories from different classes by sharing a same hidden state space. It can also automatically determine the available states and the corresponding transition matrices for one trajectory by the introducing state selection matrix *F*. However, in the sampled BP-HMM, the state transition matrix and initial probabilities are trajectory-specific, and it is not appropriate to perform mean operation on these transition matrices and probabilities to obtain a average matrix and probabilities for each class.

In order to model trajectories from different classes more reasonably, we introduce a shared hidden state space and class-specific indicators. We define a state selection vector $f_{c}$ for each class which are used to distinguish the differences between classes and define state initial probabilities $\pi_{0}$ and transition matrix $\pi_{j}$ for each class which are used to capture the commonness with one class. The transition matrix of the *c*th class from state *j* is

(13) $$\pi_{j}^{\left(c\right)}∼Dir\left(\left[r,r,\ldots ,r+\kappa ,r,\cdots \right]⊙f_{c}\right),j>0,$$

and the initial state probability vector $\pi_{0}^{\left(c\right)}$ is also related to *F*,

(14) $$\pi_{0}^{\left(c\right)}∼Dir\left(\left[r,r,\ldots ,r,r,\cdots \right]⊙f_{c}\right).$$

Similarly to the standard HMM, the latent variable $z^{\left(n\right)}$ for the *n*th trajectory is a discrete sequence with

(15) $$z_{1}^{\left(n\right)}∼\pi_{0}^{\left(y_{n}\right)},\;z_{t+1}^{\left(n\right)}|z_{t}^{\left(n\right)}∼\pi_{z_{t}^{\left(n\right)}}^{\left(y_{n}\right)},\;t=1,\ldots ,T.$$

where $y_{n}$ denotes the class of *n*th trajectory.

From the way of modeling, the proposed new version of the BP-HMM is different from the sampled BP-HMM [1] which learns an HMM for each trajectory, and it is also different from the traditional HMMs which learn an HMM for each class separately. The proposed BP-HMM can use all the sequences from different classes to jointly train a whole BP-HMM with each HMM corresponding to one class. Therefore, the proposed BP-HMM can better model the trajectories from multiple classes and can further make better classification.

### 4.2. A Simpler Representation for Beta Process

Besides the model assumption, the proposed variational BP-HMM has different representation of the BP. As introduced in Section 2, the IBP construction of the BP describes the process by conditional distributions. This kind of representation is only suitable for sampling methods which are similar to the Chinese restaurant construction of DPs. Therefore, different from the sampled BP-HMM which uses the IBP construction for the BP to lend it to a Gibbs sampler, we use the stick-breaking construction for the BP to adapt to variational inference. There is some work in constructing stick-breaking representation of BPs for variational inference. The stick-breaking construction is used for the IBP which is closely related to the BP and can be seen as a one-parameter BP [26]. The two-parameter BP is also constructed through stick-breaking processes to server for variational inference [29]. Recently, a simpler representation of the two-parameter BP based on stick-breaking construction is developed to make simpler variational inference [33]. In order to approximate posterior inference to the BP with variational Bayesian method more easily, we refer to the simpler representation of the BP [33]. Let $d_{k}$ mark the round in which the kth atom appears. That is,

(16) $$\begin{array}{c} \begin{array}{cc} & d_{k}=1+\sum_{i=1}^{\infty }\delta \left(\sum_{j=1}^{i}C_{j}<k\right). \end{array} \end{array}$$

Note $\delta \left(\cdot \right)$ is a binary indicator and it equals to 1 if the formula is true. Using the latent indicators, the representation of *B* in (6) is simplified as

(17) $$\begin{array}{c} \begin{array}{cc} & B=\sum_{k=1}^{\infty }V_{k_{d_{k}}}\prod_{l=1}^{d_{k}-1}\left(1-V_{k_{l}}\right)\delta_{\omega_{k}}, \end{array} \end{array}$$

with ω and *V* drawn as before.

Let $T_{k}=-\sum_{l<d_{k}}\ln\left(1-V_{k_{l}}\right)$. Since each individual term $-\ln\left(1-V_{k_{l}}\right)\overset{iid}{∼}\mathrm{Exponential}\left(\alpha \right)$, it follows that $T_{k}\overset{iid}{∼}\mathrm{Gamma}\left(d_{k}-1,\alpha \right)$. This gives the following representations of the BP,

(18) $$\begin{array}{c} \begin{array}{cc} & B=\sum_{k=1}^{\infty }V_{k}e^{-T_{k}}\delta_{\omega_{k}},\\ & V_{k}\overset{i.i.d.}{∼}\mathrm{Beta}\left(1,\alpha \right),\\ & T_{k}∼\mathrm{Gamma}\left(d_{k}-1,\alpha \right),\\ & \sum_{k=1}^{\infty }1\left(d_{k}=r\right)\overset{i.i.d.}{∼}\mathrm{Poisson}\left(\gamma \right),r\in N^{+},\\ & \omega_{k}\overset{i.i.d.}{∼}\frac{B_{0}}{\gamma }. \end{array} \end{array}$$

Here we should notice that each $d_{k}$ does not have a distribution, but the cardinality of $\left\{d_{k}=r\right\}$ is drawn by $\mathrm{Poisson}\left(\gamma \right)$. In addition, $T_{k}=0$ with probability one when $d_{k}=1$. In this BP, the atom $\omega_{k}=\left\{\mu_{k},\Sigma_{k}\right\}$ and Gamma priors with hyper-parameters $\left\{a_{1},a_{2}\right\}$, $\left\{b_{1},b_{2}\right\}$ are given to α and γ:

(19) $$\begin{array}{c} \begin{array}{cc} & \alpha ∼\mathrm{Gamma}\left(a_{1},a_{2}\right),\\ & \gamma ∼\mathrm{Gamma}\left(b_{1},b_{2}\right). \end{array} \end{array}$$

### 4.3. Joint Distribution of the Proposed BP-HMM

Assume that the total class number is *C* and the trajectory number is *N*. Let *X* represent the data, W={α, γ,$\{\mu_{k}$, $\Sigma_{k}\}$, $\left\{d_{k}\right\}$, $\left\{V_{k}\right\}$, $\left\{T_{k}\right\}$, $\left\{f_{ck}\right\}$, $\left\{\pi_{k}^{\left(c\right)}\right\}$, Z} represents the set of all latent variables in the model, including θ which is the set of all the hyper-parameters, and *Y* is the set of all the class labels.

**Figure 2:**
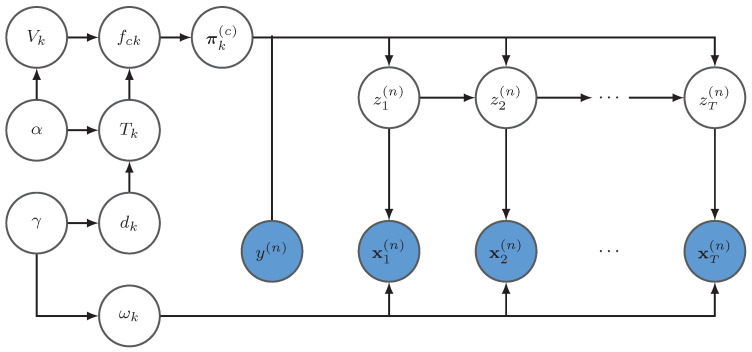
This is the probabilistic graphical model of the proposed variational BP-HMM. $X^{\left(n\right)}=\left\{x_{1}^{\left(n\right)},x_{2}^{\left(n\right)},\cdots ,x_{T}^{\left(n\right)}\right\}$ is the *n*th observed trajectory, $z^{\left(n\right)}=\left\{z_{1}^{\left(n\right)},z_{2}^{\left(n\right)},\cdots ,z_{T}^{\left(n\right)}\right\}$ is the hidden state sequence of the *n*th trajectory, and $y^{\left(n\right)}$ is the class label of the nth trajectory which indicates choosing the state transition probabilities from the class it belongs to. In this graphical model, we omit the hyper-parameters.

The probabilistic graphical model is shown in Figure 2, where its joint likelihood is

(20) $$\begin{array}{c} \begin{array}{cc} & p\left(X,W|\theta \right)=p\left(X|W,\theta \right)\times p\left(W|\theta \right). \end{array} \end{array}$$

The likelihood $p\left(X|W,\theta \right)$ is defined as a multi-normal distribution by

(21) $$p\left(X|W,\theta \right)=\prod_{n=1}^{N}\prod_{t=1}^{T}\prod_{k=1}^{K}N{\left(x_{t}^{\left(n\right)}|\mu_{k},\Sigma_{k}\right)}^{\delta (z_{t}=k)}.$$

The prior distribution of the parameter *W* and detailed setup are expressed in Appendix A.

### 4.4. Variational Inference for the Proposed BP-HMM

We use a factorized variational distribution over all the latent variables to approximate the intractable posterior $p\left(W|X,\theta \right)$. Two truncations are set in the inference: one is truncation of the number of hidden states at *K* and the other is the truncation of the round number at *R*. Specifically, we assume the variational distribution as

(22) $$\begin{array}{c} \begin{array}{cc} Q= & q\left(\alpha \right)q\left(\gamma \right)\prod_{k=1}^{K}\{q\left(\mu_{k},\Sigma_{k}\right)q\left(d_{k}\right)q\left(V_{k}\right)q\left(T_{k}\right)\\ & \left.\times \prod_{c=1}^{C}q\left(f_{ck}\right)q\left(\pi_{k}^{\left(c\right)}\right)\right\}\prod_{c=1}^{C}q\left(\pi_{0}^{\left(c\right)}\right)\prod_{n=1}^{N}q\left(z^{\left(n\right)}\right), \end{array} \end{array}$$

where

$$\begin{array}{ccc} & & q\left(\alpha \right)=\mathrm{Gamma}\left(\alpha |k_{1},k_{2}\right),\\ & & q\left(\gamma \right)=\mathrm{Gamma}\left(\gamma |\tau_{1},\tau_{2}\right),\\ & & q\left(\mu_{k},\Sigma_{k}\right)=\mathrm{NIW}\left(\mu_{k},\Sigma_{k}|u_{k},\lambda_{k},\Phi_{k},\upsilon_{k}\right),\\ & & q\left(d_{k}\right)=\mathrm{MultiNomial}\left(d_{k}|\varphi_{k}\right),\\ & & q\left(V_{k}\right)=\mathrm{Beta}\left(V_{k}|\tau_{k_{1}},\tau_{k_{2}}\right),\\ & & q\left(T_{k}\right)=\mathrm{Gamma}\left(T_{k}|u_{k}^{\prime },v_{k}^{\prime }\right),\\ & & q\left(f_{ck}\right)=\mathrm{Bernoulli}\left(f_{ck}|\upsilon_{ck}\right),\\ & & q\left(\pi_{k}^{\left(c\right)}\right)=\mathrm{Dir}\left(\pi_{k}^{\left(c\right)}|{r_{k1}^{\prime }}^{\left(c\right)},{r_{k2}^{\prime }}^{\left(c\right)},\cdots ,{r_{kK}^{\prime }}^{\left(c\right)}\right),\\ & & q\left(z^{\left(n\right)}|y_{n}\right)=\prod_{t=1}^{T}\prod_{k_{1}=1}^{K}\prod_{k_{2}=1}^{K}a_{k_{1}k_{2}}^{{\left(y_{n}\right)}^{\delta \left(z_{t}^{\left(n\right)}=k_{1},z_{t+1}^{\left(n\right)}=k_{2}\right)}}\\ & & \times \prod_{k=1}^{K}{a_{0k}^{\left(y_{n}\right)}}^{\delta \left(z_{0}^{\left(n\right)}=k\right)}\prod_{t=1}^{T}\prod_{k=1}^{K}b_{tk}^{{\left(y_{n}\right)}^{\delta \left(Z_{t}^{\left(n\right)}=k\right)}}. \end{array}$$

It is obvious that $V_{k}$ and $T_{k}$ do not have conjugate posterior. Thus the distributions are selected for better accuracy and more convenience. Here $a_{0k}^{*}$ is an estimation of the probability of the initial state distribution, $a_{j_{1j_{2}}}^{*}$, where $j_{1}>0$ and $j_{2}>0$ is an estimation of the probability of transition from state $j_{1}$ to $j_{2}$ and $b^{*}t_{j}$ is an estimation of the emission probability density given the system in state *j* at time point *t*. In order to simplify our representation, we do not use sub-index. Here $a_{i}=\left\{a_{ij}\right\}$, j=1,…,K. Let ϕ be the set of variational parameters. We expand the lower bound as $L\left(X,\phi \right)=E_{Q}\left(\ln P\left(X,W|\theta \right)\right)-E_{Q}\left[\ln Q\right]$ which is expressed in detail in Appendix B.

### 4.5. Parameter Update

In the framework of variational mean field approximation, the parameters of some variational distributions can be analytically solved using

(23) $$\ln q\left(w_{j}\right)=E_{q\left(W\neq w_{j}\right)}\left[\ln p\left(X,W|\theta \right)\right]+\mathrm{const}.$$

However, in some cases that the prior distribution and posterior distribution over one latent variable are not conjugate, the variational distribution over this variable cannot have an analytical solution. The parameters of this variational distribution should be optimized through gradient based methods with the variational lower bound being the objective.

In our model, the variational distributions q(α), q(γ), $q(\mu_{k},\Sigma_{k})$, $q(d_{k})$, $q(\pi_{k})$, q(Z) have a closed form solution, and we can get their parameter update formulas according to (23). While the variational distributions $q(V_{k})$, $q(T_{k})$, $q(f_{ck})$ cannot be analytically solved, we can update their parameters by corresponding gradients. Next, we give the way of calculating variational distributions and show the procedure for training the variational BP-HMM in Algorithm 1. The detailed parameters update formulas or the gradients with respect to the parameters are presented in Appendix C.
**Algorithm 1** Variational Inference for the Proposed BP-HMM  1:Initialize θ and ϕ.  2:Given R and threshold and Initialize RunTime = 0;  3:**while**$|L-L_{\mathrm{old}}|<\mathrm{threshold}$ or RunTime < R **do**  4: $L_{\mathrm{old}}=L$  5: **for** each trajectory *n* **do**  6:  Update $q(z^{\left(n\right)})$  7:  Calculate $q(z_{t}^{\left(n\right)}=k)$ and $q(z_{t}^{\left(n\right)}=k_{1},z_{t+1}^{\left(n\right)}=k_{2})$  8: **end for**  9: **for** each class *c* **do**10:  Update each $q(\pi_{k}^{\left(c\right)})$, k=0,…,K11:  Update each $q(f_{ck})$, k=1,…,K12: **end for**13: **for** each k=1,…,K **do**14:  Update $q(\mu_{k},\Sigma_{k})$, $q(d_{k})$, $q(T_{k})$, $q(V_{k})$15: **end for**16: Update q(α), q(γ) Calculate L17:**end while**

#### 4.5.1. Calculation for q(α), q(γ), $q(\mu_{k},\Sigma_{k})$, $q(d_{k})$, $q(\pi_{k}^{\left(c\right)})$, q(Z)


$$\begin{array}{ccc} & & \ln q\left(\alpha \right)=E_{q}\left[\ln p\left(\alpha \right)+\sum_{k=1}^{K}\ln p\left(V_{k}|\alpha \right)+\ln p\left(T_{k}|d_{k},\alpha \right)\right],\\ & & \ln q\left(\gamma \right)=E_{q}\left[\ln p\left(\gamma \right)+\sum_{k=1}^{K}\ln p\left(d_{k}|\gamma \right)\right],\\ & & \ln q\left(\mu_{k},\Sigma_{k}\right)=E_{q}[\ln p\left(\mu_{k},\Sigma_{k}|\theta \right)\\ & & \;\;\;\;\;\;\;\;\;\;\;\;\;\;\;\;\;\;\;\;\;\;+\sum_{n=1}^{N}\sum_{t=1}^{T}p\left(x^{\left(n\right)}|z^{\left(n\right)},\mu_{k},\Sigma_{k}\right)],\\ & & \ln q\left(d_{k}\right)=E_{q}[\ln p\left(d|\gamma \right)+\ln p\left(T_{k}|d_{k},\alpha \right)\\ & & \;\;\;\;\;\;\;\;\;\;\;\;\;\;\;\;\;+\sum_{c=1}^{C}\ln p\left(f_{ck}|V_{k},T_{k},d_{k}\right)],\\ & & \ln q\left(\pi_{k}^{\left(c\right)}\right)=E_{q}[\ln p\left(\pi_{k}^{\left(c\right)}|f_{ck},r,\kappa \right)\\ & & \;\;\;\;\;\;\;\;+\sum_{n=1}^{N}\sum_{t=1}^{T-1}\delta \left(y_{n}=c\right)\ln p\left(z_{t+1}^{\left(n\right)}|\pi_{k}^{\left(c\right)},z_{t}^{\left(n\right)}=k\right)],\\ & & \ln q\left(z^{\left(n\right)}\right)=E_{q}[\ln p\left(x^{\left(n\right)}|z^{\left(n\right)},\mu_{k},\Sigma_{k}\right)\\ & & \;\;\;\;\;\;\;\;\;\;\;\;\;\;\;\;\;\;\;+\sum_{n=1}^{N}\ln p\left(z^{\left(n\right)}|\Pi^{\left(y_{n}\right)}\right)], \end{array}$$

#### 4.5.2. Optimization for $q(V_{k})$, $q(T_{k})$, $q(f_{ck})$

The variational parameters of $q(V_{k})$, $q(T_{k})$, $q(f_{ck})$ include $\left\{\tau_{k_{1}},\tau_{k_{2}}\right\}$, $\left\{u_{k}^{\prime },v_{k}^{\prime }\right\}$, $\left\{\upsilon_{ck}\right\}$. They are updated by the gradient based method where the gradients of the lower bound L with respect to these parameters should be calculated.

#### 4.5.3. Remarks

Note that when updating the BP parameters, we should calculate the expectation as

$$\begin{array}{ccc} E_{q}\left[\ln p\left(f_{ck}|V_{k},T_{k}\right)\right] & = & v_{ck}E_{q}\left[\ln V_{k}e^{-T_{k}}\right]\\ & & +\left(1-v_{ck}\right)E_{q}\left[1-\ln V_{k}e^{-T_{k}}\right], \end{array}$$

of which the second term is intractable. We refer the work in [33] to use a Taylor expansion to $E_{q}\left[\ln\left(1-V_{k}e^{-T_{k}}\right)\right]$ about the point one,

(24) $$E_{q}\left[\ln\left(1-V_{k}e^{-T_{k}}\right)\right]=-\sum_{m=1}^{M}\frac{1}{m}{\left(V_{k}e^{-T_{k}}\right)}^{m}.$$

For clarity, we define each term $\frac{1}{m}E\left[{\left(V_{k}e^{-T_{k}}\right)}^{m}\right]$ in the Taylor expansion using the notation $\Delta_{k}\left(m\right)$ as

$$\begin{array}{c} \begin{array}{cc} \Delta_{k}\left(m\right) & =\frac{1}{m}\frac{\Gamma \left(\tau_{k_{1}}+\tau_{k_{2}}\right)}{\Gamma \left(\tau_{k_{1}}+\tau_{k_{2}}+m\right)}\frac{\Gamma \left(\tau_{k_{1}}+m\right)}{\Gamma \left(\tau_{k_{1}}\right)}{\left(\frac{v_{k}^{\prime }}{v_{k}^{\prime }+m}\right)}^{u_{k}^{\prime }}\\ & =\prod_{i=1}^{m}\frac{\tau_{k_{1}}+i-1}{\tau_{k_{1}}+\tau_{k_{2}}+i-1}{\left(\frac{v_{k}^{\prime }}{v_{k}^{\prime }+m}\right)}^{u_{k}^{\prime }}, \end{array} \end{array}$$

and define $\Delta_{k}\left(\cdot \right)=\sum_{m=1}^{M}\Delta_{k}\left(m\right)$. Therefore, $E_{q}\left[\ln\left(1-V_{k}e^{-T_{k}}\right)\right]=-\Delta_{k}\left(\cdot \right)$.

### 4.6. Classification

Our model is applicable to trajectory recognition like human activity trajectory recognition. We use the proposed variational BP-HMM to model all the training data from different classes, with each HMM corresponding to a class. Given the learned model with the hyperparameters and variational parameters {θ,ϕ}, a new test trajectory $x^{*}$ can be classified according to its marginal likelihood $p(x^{*}|\theta ,\phi )$. Denote $y^{*}$ as the label of the test trajectory; the classification criteria can be expressed as

(25) $$\begin{array}{c} \begin{array}{cc} y^{*} & =\underset{c}{\arg\max}\ln p\left(x^{*}|\left\{{a_{k}^{*}}^{\left(c\right)},u_{k},\lambda_{k},\upsilon_{k},\Phi_{k}\right\},{a_{0}^{*}}^{\left(c\right)}\right),\\ & =\underset{c}{\arg\max}\ln(\int p\left(x^{*}|\left\{\mu_{k},\Sigma_{k}\right\},z\right)p\left(z|\left\{{a_{k}^{*}}^{\left(c\right)}\right\}\right)\\ & \;\;\;\;\;\;\prod_{k=1}^{K}p\left(\mu_{k},\Sigma_{k}|u_{k},\lambda_{k},\upsilon_{k},\Phi_{k}\right)dzd\mu_{k}d\Sigma_{k}), \end{array} \end{array}$$

where ${a_{jk}^{*}}^{\left(c\right)}$ is an estimate of the probability of transition from state *j* to *k* in the *c*th class. The likelihood can be calculated through the forward-backward algorithm.

This classification mechanism is more reasonable than the method in [1], as the transition matrix is actually learned.

## 5. Experiment

To demonstrate the effectiveness of our model on trajectory recognition, we conduct experiments on one synthetic dataset and two real-world datasets; the detailed data statistics are illustrated in Table 1 and the following subsections. We compare our model with HCRF, LSTM [34], HMM-BIC and the sampled BP-HMM. In particular, in HCRF, the number of hidden states is set to 15 and the size of the window is set to 0. In LSTM, we use a recurrent neural network with one hidden layer as its architecture. In HMM-BIC, the state number is selected from the range [1,20]. In the sampled BP-HMM, the hyperparameters are set according to Sun et al. [1]. In the variational BP-HMM, the hyperparameters $\left\{a_{1},a_{2},b_{1},b_{2},r,\kappa \right\}$ are randomly initialized and selected by maximizing the variational lower bound, and the emission hyperparameters are initialized with k-means. Particularly, the state truncation parameters in variational BP-HMM are set according to specific datasets, e.g., K=7 for the synthetic data and K=20 for the two real-world data. All experiments are repeated ten times with different training and test division methods, and the average classification accuracy with the standard deviation is reported.

### 5.1. Synthetic Data

The synthetic data called control chart patterns (CCP) have some quantifiable similarities. They contain four pattern types which can be downloaded from the UCI machine learning repository. The CCP are trajectories that show the level of a machine parameter plotted against time. 400 trajectories are artificially generated by the following four equations [35]:

$$\begin{array}{c} \begin{array}{cc} & 1.\;\mathrm{Normal}\;\mathrm{pattern}\;(\mathrm{Normal}):\;y\left(t\right)=m+rs,\\ & \;\mathrm{where}\;m=3,s=2\;\mathrm{and}\;0<r<1.\\ & 2.\;\mathrm{Cyclic}\;\mathrm{pattern}\;(\mathrm{Cyclic}):\;y\left(t\right)=m+rs+a\mathrm{Sin}\left(2\pi t/T\right),\\ & \;\mathrm{where}\;0<a,T<15.\\ & 3.\;\mathrm{Increasing}\;\mathrm{trend}\;(\mathrm{IT}):\;y\left(t\right)=m+rs+gt,\\ & \;\mathrm{where}\;0.2<g<0.5.\\ & 4.\;\mathrm{Decreasing}\;\mathrm{trend}\;(\mathrm{DT}):\;y\left(t\right)=m+rs-gt,\\ & \;\mathrm{where}\;0.2<g<0.5. \end{array} \end{array}$$

**Figure 3:**
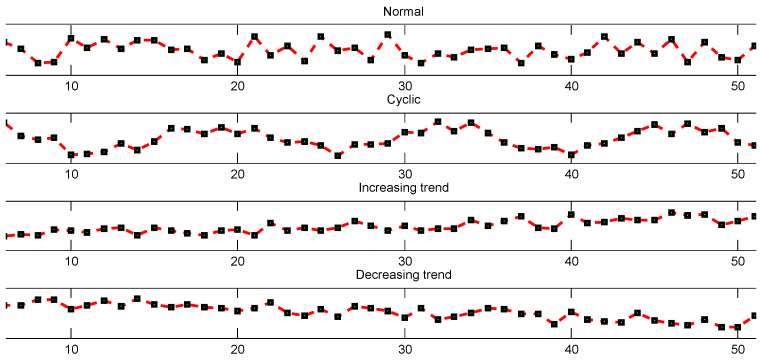
Examples of control chart patterns.

**Figure 4:**
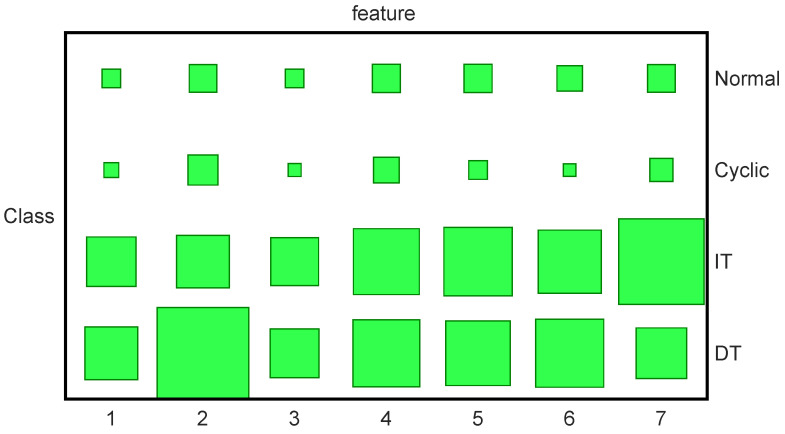
Selection results of hidden states for four classes on control chart patterns; these four classes are normal pattern (Normal), cyclic pattern (Cyclic), increasing trend (IT) and decreasing trend (DT). The occurrence probabilities $q(f_{ck})$ of the hidden states are presented by the sizes of the green blocks. The large size of the green blocks represents high occurrence probability of hidden states.

Figure 3 shows the generated synthetic data. In this experiment, 20 trajectories are used for training with 5 trajectories for each class. The classification results are presented in Table 2. The results are obtained through five-fold cross-validation. In order to illustrate that the sharing patterns have been learned by our method, the Hinton diagrams of the variational parameter *V* are given in Figure 4, where the occurrence probabilities of the hidden states are presented by the sizes of the blocks. For example, we can find that IT and DT share the 4th, 5th, 6th features.

We compare our method with HCRF, LSTM, HMM-BIC and the sampled BP-HMM. As we can see from Table 2, our method outperforms all the other methods.

In this experiment, the sharing patterns contribute to improving the performance. Since an HMM is created for each class of trajectories in our proposed method instead of each trajectory in the sampled BP-HMM, our method has better performance than the sampled BP-HMM.

### 5.2. Human Activity Trajectory Recognition

**Figure 5:**
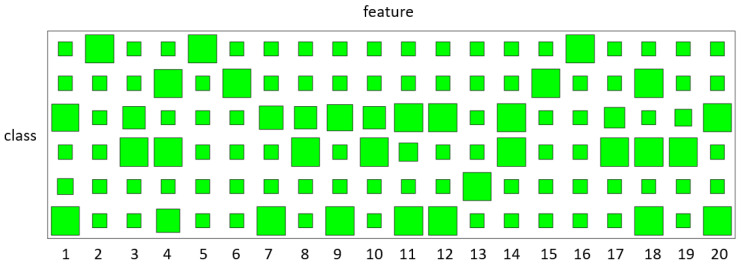
Selection results of hidden states for different classes on human activity trajectory recognition. The occurrence probabilities $q(f_{ck})$ of the hidden states are presented by the sizes of the green blocks. The large size of the green blocks represents high occurrence probability of hidden states.

**Figure 6:**
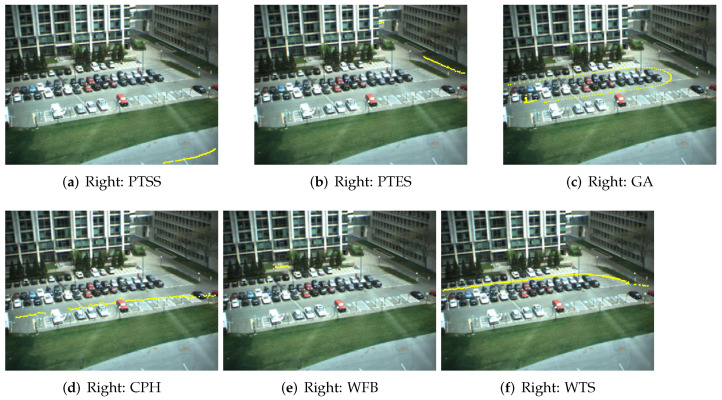
Correct classification results of HATR dataset for the classes: PTSS, PTES, GA, CPH, WFB, WTS, respectively.

**Figure 7:**
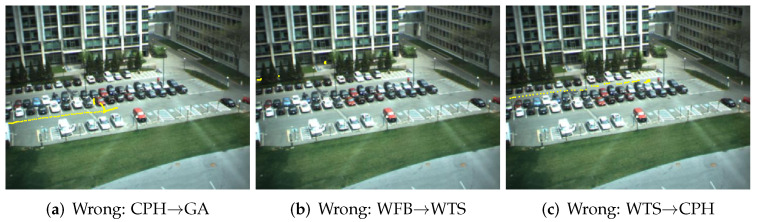
Misclassification results of HATR dataset for the three classes: CPH, WFB and WTS which are misclassified to GA, WTS and CPH, respectively.

Human activity trajectory recognition (HATR) [36] is important in many applications such as health care. In our human activity trajectory recognition experiment, parking lot data are collected from the video [1]. We use the data tagged manually [1], which has 300 trajectories with 50 trajectories for each class. Six classes are defined, which are “passing through south street” (PTSS), “passing through east street” (PTES), “going around” (GA), “crossing park horizontally” (CPH), “wandering in front of building” (WFB) and “walking in top street” (WTS). As seen from [1], the sampled BP-HMM is the best method among the methods including HCRF, LSTM, HMM-BIC and the sampled BP-HMM in HATR. Here we use the same training and test data to compare the variational BP-HMM with the sampled BP-HMM. Table 3 shows the comparisons of the classification accuracy for the proposed method VBP-HMM versus HCRF, LSTM, HMM-BIC and the sampled-BP-HMMs in HATR. The results are obtained through five-fold cross-validation. As can be seen from Table 3, the accuracy of our method is 0.96, while the accuracy of the sampled BP-HMM is 0.91 [1]. The detailed confusion matrix for our method is given in Table 4. The state sharing patterns learned by variational BP-HMM are displayed with the Hinton diagrams in Figure 5, in which GA and CPH, as well as GA and WTS, are more likely to share states. The good performance verifies the superiority of modeling an HMM for each class. Moreover, we take some examples of the correct classification and misclassification results for visualization as in Figure 6 and Figure 7. As illustrated in Figure 7, the misclassified trajectories often contain some deceptive subpatterns such as the trajectory of CPH in subfigure (d) containing a back turn and a left turn like the GA class.

### 5.3. Wall-Following Navigation Task

**Figure 8:**
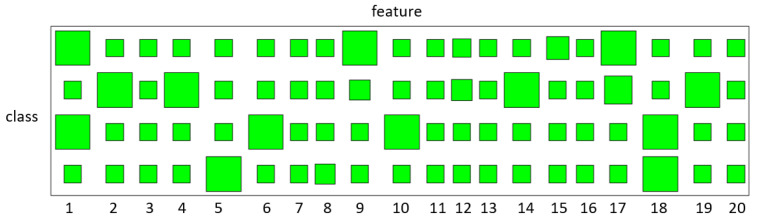
Selection results of hidden states for different classes on the Wall-Following navigation recognition. The occurrence probabilities $q(f_{ck})$ of the hidden states are presented by the sizes of the green blocks. The large size of the green blocks represents high occurrence probability of hidden states.

We perform the Wall-Following navigation task (WFNT) in which data are collected from the sensors on the mobile robot SCITOS-G5 [4]. We think that this task is a trajectory with historical data, and two ultrasound sensors datasets are selected, because the cost is as low as possible in civil applications with acceptable accuracy. There are 187 trajectories in the data and four classes need to be recognized, which are “front distance” (F), “left distance” (L), “right distance” (R) and “back distance” (B). We randomly select 40 training trajectories with 10 for each class. The confusion matrix of classification is shown in Table 5 and the state sharing patterns learned by variational BP-HMM are displayed with the Hinton diagrams in Figure 8, where R and F, as well as R and B, have a small number of shared states.

The comparison of the classification accuracy for our method VBP-HMM versus HCRF, LSTM, HMM-BIC and the sampled BP-HMM is shown in Table 6. The results are obtained by five-fold cross-validation. It is obvious that our method is much better than the sampled BP-HMM, because we create an HMM for each class of trajectories rather than create an HMM for each trajectory. Although the sharing patterns are not obvious in this experiment, our method has better performance than the other methods. As we have analyzed, sharing patterns among different classes will be learned automatically by our model, which helps to localize precisely the difference of different classes. When there is no sharing pattern among classes, the advantage will be weakened.

### 5.4. Performance Analysis

In our experiments, the results show that the proposed variational BP-HMM has a great improvement compared to the sampled BP-HMM which uses average transition over trajectories from each class. We analyze the advantages of variational BP-HMM for the following reasons. Due to the small amount of training data in our experiment, the performance of LSTM is not satisfactory. HMM-BIC finds an optimal state number through model selection but it cannot make use of the shared information among classes, and its performance is the second-best overall. Although the sample BP-HMM can share hidden states among classes, it does not make correct use of the shared information in classification and thus does not gain better results. Our proposed variational BP-HMM constructs a mechanism to learn shared hidden states by introducing state indicator variables and maintains class-specific state transition matrices which are very helpful for classification tasks.

Moreover, we give the total cost time of the variational BP-HMM, HMM-BIC, LSTM, HCRF and the sampled BP-HMM in Table 7, where we can see the variational BP-HMM performs much more efficiently than the sampled BP-HMM. This is attributed to the efficiency of the variational methods. Although the sampled BP-HMM and the variational BP-HMM have similar time complexity, due to the sampling operation, the cost time of the sampled BP-HMM is usually several times that of the variational BP-HMM. In other words, the variational BP-HMM converges much faster than the sampled BP-HMM. Besides, compared with HMM-BIC, it only takes about twice the time to achieve significant performance improvements. Above all, we can conclude that the proposed variational BP-HMM is an effective and efficient method for trajectory recognition.

## 6. Conclusions

In this paper, we have proposed a novel variational BP-HMM for modeling and recognizing trajectories. The proposed variational BP-HMM has shared hidden state space which is used to capture the commonality of the cross-category data and class-specific indicators which are used to distinguish the data from different classes. As a result, in the variational BP-HMM, multiple HMMs are used to model multiple classes of trajectories among which a hidden state space is shared.

The more reasonable assumptions of the proposed model make it more suitable for jointly modeling trajectories over all classes and further making trajectory recognition. Experimental results both on synthetic and real-world data have verified that the proposed variational BP-HMM can find the feature sharing patterns among different classes, which helps to better model trajectories and further improve the classification performance. Moreover, compared with the sampled BP-HMM, the derived variational inference for the proposed BP-HMM can reduce the time cost of the training procedure. The experimental time records also show the efficiency of the proposed variational BP-HMM.

## Figures and Tables

**Table 1:** Data statistics for the CCP, HATR and WFNT datasets and corresponding classes.

Datasets	#Train Trajectories	#Classes (Descriptions)
CCP	20 (5/class)	4 (Normal, Cyclic, IT, DT)
HATR	300 (50/class)	6 (PTSS, PTES, GA, CPH, WFB, WTS)
WFNT	40 (10/class)	4 (F, L, R, B)

**Table 2:** Comparisons of the classification accuracy for the proposed method VBP-HMM versus HCRF, LSTM, HMM-BIC and the sampled BP-HMM in CCP.

	Classification Accuracy				
Approach	HCRF	LSTM	HMM-BIC	SBP-HMM	VBP-HMM
CCP	0.88 ± 0.03	0.95 ± 0.02	0.97 ± 0.01	0.96 ± 0.02	1.00 ± 0.00

**Table 3:** Comparisons of the classification accuracy for the proposed method VBP-HMM versus HCRF, LSTM, HMM-BIC and the sampled BP-HMM in HATR.

	Classification Accuracy				
Approach	HCRF	LSTM	HMM-BIC	SBP-HMM	VBP-HMM
HATR	0.68 ± 0.03	0.75 ± 0.03	0.95 ± 0.02	0.91 ± 0.02	0.96 ± 0.02

**Table 4:** Classification accuracy for human activity trajectory recognition.

	Classification Accuracy					
Predicted Class	PTSS	PTES	GA	CPH	WFB	WTS
PTSS	1.00	0.00	0.00	0.00	0.00	0.00
PTES	0.00	1.00	0.00	0.00	0.00	0.00
GA	0.00	0.00	0.97	0.00	0.00	0.00
CPH	0.00	0.00	0.03	0.96	0.00	0.00
WFB	0.00	0.00	0.00	0.03	1.00	0.23
WTS	0.00	0.00	0.00	0.01	0.00	0.77

**Table 5:** The confusion matrix of the Wall-Following navigation recognition.

	Classification Accuracy			
Predicted Class	F	L	R	B
F	0.95	0.18	0.00	0.00
L	0.02	0.73	0.00	0.00
R	0.03	0.09	0.95	0.0
B	0.00	0.00	0.05	1.00

**Table 6:** Comparisons of the classification accuracy for the proposed method VBP-HMM versus HCRF, LSTM, HMM-BIC and the sampled BP-HMM in WFNT.

	Classification Accuracy				
Approach	HCRF	LSTM	HMM-BIC	SBP-HMM	VBP-HMM
WFNT	0.80 ± 0.03	0.73 ± 0.08	0.86 ± 0.04	0.85 ± 0.02	0.89 ± 0.01

**Table 7:** Comparisons of total time cost for the proposed method VBP-HMM versus HCRF, LSTM, HMM-BIC and the sampled-BP-HMM in experiments.

	Total Time Cost (s)				
Approach	HCRF	LSTM	HMM-BIC	SBP-HMM	VBP-HMM
CCP	196	6	54	2151	117
HATR	118	13	93	2521	205
WFNT	1005	8	115	1819	312

## Data Availability

Data available in a publicly accessible repository. The data presented in this study are openly available in UCI Machine Learning Repository at http://archive.ics.uci.edu/ml/index.php, accessed on 27 September 2021, reference number [4,35,36].

## Appendix A. The Prior Distribution of the Parameter *W*

Denote $\sum_{k=1}^{\infty }\delta \left(d_{k}=r\right)$ as *d*. The prior distribution of the parameter *W* is expressed as

(A1) $$\begin{array}{c} \begin{array}{cc} p\left(W|\theta \right) & =p\left(\alpha \right)p\left(\gamma \right)p\left(d|\gamma \right)\\ & \times \prod_{k=1}^{\infty }\{p\left(V_{k}|\alpha \right)p\left(T_{k}|d_{k},\theta \right)p\left(\mu_{k},\Sigma_{k}|\theta \right)\\ & \;\;\;\times \prod_{c=1}^{C}p\left(f_{c_{k}}|V_{k},T_{k},d_{k}\right)p\left(\pi_{k}^{\left(c\right)}|f_{c},\theta \right)\}\\ & \times \prod_{c=1}^{C}p\left(\pi_{0}^{\left(c\right)}|f_{c},\theta \right)\\ & \times \prod_{n=1}^{N}\{\prod_{t=1}^{T}\prod_{k_{1}=1}^{\infty }\prod_{k_{2}=1}^{\infty }{\left(\pi_{k_{1}k_{2}}^{\left(y_{n}\right)}\right)}^{\delta \left(z_{t}^{\left(n\right)}=k_{1},z_{t+1}^{\left(n\right)}=k_{2}\right)}\\ & \;\;\;\times \prod_{k=1}^{\infty }{\left(\pi_{0k}^{\left(y_{n}\right)}\right)}^{\delta \left(z_{0}^{\left(n\right)}=k\right)}\}. \end{array} \end{array}$$

We use

$$p\left(f_{c_{k}}|V_{k},T_{k},d_{k}\right)=p{\left(f_{c_{k}}|V_{k}\right)}^{\delta \left(d_{k}=1\right)}p{\left(f_{c_{k}}|V_{k},T_{k}\right)}^{\delta \left(d_{k}>1\right)}$$

to account for the class in which an atom appears. Two terms $p\left(T_{k}|d_{k},\alpha \right)$ and $p\left(d|\gamma \right)$ are given by Paisley et al. [33],

$$p\left(T_{k}|d_{k},\alpha \right)=\frac{\alpha^{\nu_{k}\left(\delta \right)}}{\prod_{r⩾2}\Gamma {\left(r-1\right)}^{1\left(d_{k}=r\right)}}T_{k}^{\nu_{k}\left(2\right)}e^{-\alpha T_{k}\delta \left(d_{k}>1\right)},$$

where $\nu_{k}\left(s\right)=\sum_{r⩾2}\left(r-s\right)e^{-\alpha T_{k}\delta \left(d_{k}=r\right)}$, and

$$p\left(d|\gamma \right)=\prod_{r=1}^{\infty }\frac{\gamma \sum_{k}\delta \left(d_{k}=r\right)}{\sum_{k}\delta \left(d_{k}=r\right)!}e^{-\gamma \left(\sum_{r^{{}^{\prime }}=r}^{\infty }\sum_{k=1}^{\infty }\delta \left(\delta \left(d_{k}=r^{{}^{\prime }}\right)>0\right)\right)}.$$

In $p\left(T_{k}|d_{k},\alpha \right)$, the indicator $d_{k}$ is used for selecting the Gamma prior parameters of $T_{k}$. Moreover, the term $p\left(T_{k}|d_{k},\alpha \right)$ in (21) will be removed if $d_{k}=1$. The binary indicator $\delta \left(\sum_{r^{{}^{\prime }}=r}^{\infty }\sum_{k=1}^{\infty }\delta \left(\delta \left(d_{k}=r^{\prime }\right)>0\right)\right)$ in $p\left(d|\gamma \right)$ means that at least one of the *K* indexed atoms occur in round *r* or the round after *r*.

## Appendix B. The Lower Bound $L\left(X,\phi \right)$

We expand the lower bound as $L\left(X,\phi \right)=E_{Q}\left(\ln P\left(X,W|\theta \right)\right)-E_{Q}\left[\ln Q\right]$ which is expressed as

$$\begin{array}{ccc} & & L\left(X,\phi \right)\\ & & =\sum_{n=1}^{N}\{\sum_{t=1}^{T}\sum_{k=1}^{K}E_{q}\left[\delta \left(z_{t}^{\left(n\right)}=k\right)\ln p\left(X_{t}|\mu_{k},\Sigma_{k},\theta \right)\right]\\ & & \;+\sum_{t=1}^{T-1}\sum_{k_{1}=1}^{K}\sum_{k_{2}=1}^{K}E_{q}\left[\delta \left(z_{t}^{\left(n\right)}=k_{1},z_{t+1}^{\left(n\right)}=k_{2}\right)\ln\left(\pi_{k}^{\left(y_{n}\right)}\right)\right]\\ & & \;+\sum_{k=1}^{K}E_{q}\left[\delta \left(z_{0}^{\left(n\right)}=k\right)\ln\left(\pi_{0k}^{\left(y_{n}\right)}\right)\right]\}\\ & & +\sum_{c=1}^{C}\{\sum_{k=0}^{K}E_{q}\left[\ln(p\left(\pi_{k}^{\left(c\right)}|f_{c},\theta \right))\right]\\ & & \;+\sum_{k=1}^{K}E_{q}\left[\delta \left(d_{k}=1\right)\ln p\left(f_{ck}|V_{k}\right)\right]\\ & & \;+\sum_{k=1}^{K}E_{q}\left[\delta \left(d_{k}>1\right)\ln p\left(f_{ck}|V_{k},T_{k}\right)\right]\}\\ & & +\sum_{k=1}^{K}\{E_{q}\ln p\left(\mu_{k},\Sigma_{k}|\theta \right)+E_{q}\left[\ln p(T_{k}|\alpha ,d_{k})\right]\\ & & +E_{q}\left[\ln p(V_{k}|\alpha )\right]\}\\ & & +\sum_{r=1}^{\infty }E_{q}\left[\ln p\left(\Sigma_{k}\delta \left(d_{k}=r\right)|\gamma \right)\right]\\ & & +E_{q}\left[\ln p\left(\alpha \right)\right]+E_{q}\left[\ln p\left(\gamma \right)\right]\\ & & -E_{Q}\left[\ln Q_{-T}\right]-\sum_{k=1}^{K}\varphi_{k}\left(r>1\right)E_{q}\left[\ln q\left(T_{k}\right)\right]. \end{array}$$

Note that we multiply the entropy of $T_{k}$, $E_{q}\left(T_{k}\right)\ln q\left(T_{k}\right)$, by the variational probability $\varphi_{k}\left(r>1\right)$ as done in [33] for keeping the entropy of $T_{k}$ from blowing up when $\varphi_{k}\left(1\right)\to 1$, where $\varphi_{k}\left(r>1\right)=\sum_{r>1}\varphi_{kr}=E_{q}\left[\delta \left(d_{k}>1\right)\right]$.

## Appendix C. Coordinate Update for the Key Distributions

### Appendix C.1. Coordinate Update for q(f_ck_)

Since the Dirichlet distribution of $\pi_{k}$, $f_{ck}$ cannot be obtained by analysis directly. The gradient ascent algorithm is used for updating $f_{ck},c\in \left\{1,\ldots ,C\right\}$. The derivative of L with respect to $\upsilon_{ck}$ is

$$\begin{array}{c} \begin{array}{cc} \frac{\partial L}{\partial \upsilon_{ck}} & =\sum_{j=0}^{K}\{\left(r^{\left(c\right)}-1\right)\left(\Psi \left(r_{jk}^{\prime (c)}\right)-\Psi \left(\sum_{t=1}^{K}r_{jt}^{\prime (c)}\right)\right)\delta \left(j\neq k\right)\\ & \left.+\left(r^{\left(c\right)}+\kappa -1\right)\left(\Psi \left(r_{jk}^{\prime (c)}\right)-\Psi \left(\sum_{k=1}^{K}r_{jk}^{\prime (c)}\right)\right)\delta \left(j=k\right)\right.\\ & +\Psi \left(\sum_{t=1}^{K}v_{ct}r^{\left(c\right)}\delta \left(t\neq j\right)+v_{cj}\left(r^{\left(c\right)}+\kappa \right)\delta \left(t=j\right)\right)\\ & \times \left(\left(r^{\left(c\right)}+\kappa \right)\delta \left(j=k\right)+r^{\left(c\right)}\delta \left(j\neq k\right)\right)\\ & -\Psi \left(v_{ck}r^{\left(c\right)}\right)r^{\left(c\right)}\delta \left(j\neq k\right)\\ & -\Psi \left(v_{ck}\left(r^{\left(c\right)}+\kappa \right)\right)\left(r^{\left(c\right)}+\kappa \right)\delta \left(j=k\right)\}\\ & +\varphi_{k}\left(1\right)\left(\Psi \left(\tau_{k_{1}}\right)-\Psi \left(\tau_{k_{2}}\right)\right)\\ & +\varphi_{k}\left(r>1\right)\left(\Psi \left(\tau_{k_{1}}\right)-\Psi \left(\tau_{k_{1}}+\tau_{k_{2}}\right)-\frac{u_{k}^{\prime }}{v_{k}^{\prime }}+{▵}_{k}\left(\cdot \right)\right)\\ & +\ln\upsilon_{ck}-\ln\left(1-\upsilon_{ck}\right). \end{array} \end{array}$$

### Appendix C.2. Coordinate Update for q(d_k_)

The update for each $\varphi_{k}$ is given below for r=1,…,R. Let

$$\begin{array}{c} \begin{array}{cc} \rho \left(r\right)= & \left(r-1\right)\left(\Psi \left(k_{1}\right)-\ln k_{2}\right)-\ln\Gamma \left(r-1\right)\\ & +\left(r-2\right)\left(\Psi \left(u_{k}^{\prime }\right)-\ln v_{k}^{\prime }\right). \end{array} \end{array}$$

If r=1,

$$\begin{array}{c} \begin{array}{cc} \varphi_{k}\left(1\right) & \propto \exp\left\{n_{0k}\left(\Psi \left(\tau_{k_{2}}\right)-\Psi \left(\tau_{k_{1}}+\tau_{k_{2}}\right)\right)-\xi \sum_{i\neq k}\varphi_{i}\left(1\right)\right\}. \end{array} \end{array}$$

If r>2,

$$\begin{array}{c} \begin{array}{cc} \varphi_{k}\left(r\right) & \propto \exp\{n_{1k}\left(\Psi \left(\tau_{k_{1}}\right)-\Psi \left(\tau_{k_{1}}+\tau_{k_{2}}\right)-\frac{u_{k}^{\prime }}{v_{k}^{\prime }}\right)\\ & -n_{0k}\Delta_{k}\left(\cdot \right)+H\left[q\left(T_{k}\right)\right]+\rho \left(r\right)\\ & -\xi \sum_{i\neq k}\varphi_{k}\left(r\right)-\frac{\tau_{1}}{\tau_{2}}\sum_{j=2}^{r}\prod_{k^{\prime }\neq k}\sum_{r^{\prime }=1}^{j-1}\varphi_{k^{\prime }}\left(r^{\prime }\right)\}. \end{array} \end{array}$$

where $n_{1k}=\sum_{c=1}^{C}v_{ck}$, $n_{0k}=C-n_{1k}$ and $\xi =\sum_{x}^{\infty }x^{-2}\ln\left(x\right)\approx 0.9375$.

### Appendix C.3. Coordinate Update for q(V_k_)

We use the gradient ascent algorithm to jointly update $\left(\tau_{k_{1}},\tau_{k_{2}}\right)$ by the gradients of the lower bound with respect to $\left(\tau_{k_{1}},\tau_{k_{2}}\right)$. Let $\lambda_{1}=-n_{0_{k}}\varphi_{k}\left(1\right)-n_{1_{k}}-\frac{k_{1}}{k_{2}}-1+\tau_{k_{1}}+\tau_{k_{2}}$, $\lambda_{2}=-n_{0_{k}}\varphi_{k}\left(r>1\right)$, $\lambda_{3}=n_{1_{k}}+1-\tau_{k_{1}}$ and $\lambda_{4}=n_{0_{k}}\varphi_{k}\left(1\right)+\frac{k_{1}}{k_{2}}-\tau_{k_{2}}$. The derivatives are

$$\begin{array}{c} \begin{array}{cc} \frac{\partial L}{\partial \tau_{k_{1}}}= & \lambda_{3}\Psi^{\prime }\left(\tau_{k_{1}}\right)+\lambda_{1}\Psi^{\prime }\left(\tau_{k_{1}}+\tau_{k_{2}}\right)+\lambda_{2}\frac{\partial \Delta_{k}\left(\cdot \right)}{\partial \tau_{k_{1}}},\\ \frac{\partial L}{\partial \tau_{k_{2}}}= & \lambda_{4}\Psi^{\prime }\left(\tau_{k_{2}}\right)+\lambda_{1}\Psi^{\prime }\left(\tau_{k_{1}}+\tau_{k_{2}}\right)+\lambda_{2}\frac{\partial \Delta_{k}\left(\cdot \right)}{\partial \tau_{k_{2}}}. \end{array} \end{array}$$

Since $\Psi \left(x\right)$ can be expanded as $\Psi \left(x\right)=-\gamma +\sum_{k=1}^{\infty }\left(\frac{1}{k}+\frac{1}{x+k}\right)$ and its derivative is $\Psi^{\prime }\left(x\right)=\sum_{k=1}^{\infty }{\left(x+k-1\right)}^{-2}$, we can get

$$\begin{array}{c} \begin{array}{cc} \frac{\partial \Delta_{k}\left(\cdot \right)}{\partial \tau_{k_{1}}}=\sum_{m=1}^{M}\{ & \frac{1}{m}{\left(\frac{v_{k}^{\prime }}{v_{k}^{\prime }+m}\right)}^{u_{k}^{\prime }}\prod_{i=1}^{m}\frac{\tau_{k_{1}}+i-1}{\tau_{k_{1}}+\tau_{k_{2}}+i-1}\\ & \times \{\Psi \left(\tau_{k_{1}}+\tau_{k_{2}}+m\right)+\Psi \left(\tau_{k_{1}}\right)\\ & -\Psi \left(\tau_{k_{1}}+\tau_{k_{2}}\right)-\Psi \left(\tau_{k_{1}}+m\right)\}\}, \end{array} \end{array}$$

and

$$\begin{array}{c} \begin{array}{cc} \frac{\partial \Delta_{k}\left(\cdot \right)}{\partial \tau_{k_{2}}}=\sum_{m=1}^{M}\{ & \frac{1}{m}{\left(\frac{v_{k}^{\prime }}{v_{k}^{\prime }+m}\right)}^{u_{k}^{\prime }}\prod_{i=1}^{m}\frac{\tau_{k_{1}}+i-1}{\tau_{k_{1}}+\tau_{k_{2}}+i-1}\\ & \times \left(\Psi \left(\tau_{k_{1}}+\tau_{k_{2}}+m\right)-\Psi \left(\tau_{k_{1}}+\tau_{k_{2}}\right)\right)\}. \end{array} \end{array}$$

### Appendix C.4. Coordinate Update for q(T_k_)

We use the gradient ascent algorithm to jointly update $\left(u_{k}^{\prime },v_{k}^{\prime }\right)$ by the gradients of the lower bound with respect to $\left(u_{k}^{\prime },v_{k}^{\prime }\right)$. The derivatives are

(A2) $$\begin{array}{c} \begin{array}{cc} \frac{\partial L}{\partial u_{k}^{\prime }}= & \Psi^{\prime }\left(u_{k}^{\prime }\right)\sum_{r>1}\left(r-2\right)\varphi_{k}\left(r\right)\\ & +\varphi_{k}\left(r>1\right)\left(1-\frac{n_{1_{k}}+\frac{k_{1}}{k_{2}}}{v_{k}^{\prime }}\right.\\ & \left.-n_{0_{k}}\frac{\partial \Delta_{k}\left(\cdot \right)}{\partial u_{k}^{\prime }}+\left(1-u_{k}^{\prime }\right)\Psi {}^{\prime }\left(u_{k}^{\prime }\right)\right),\\ \frac{\partial L}{\partial v_{k}^{\prime }}= & -\frac{1}{v_{k}^{\prime }}\sum_{r>1}\left(r-2\right)\varphi_{k}\left(r\right)+\varphi_{k}\left(r>1\right)\\ & \times \left(\frac{u_{k}^{\prime }}{{v_{k}^{\prime }}^{2}}\left(n_{1_{k}}+\frac{k_{1}}{k_{2}}\right)-n_{0_{k}}\frac{\partial \Delta_{k}\left(\cdot \right)}{\partial v_{k}^{\prime }}-\frac{1}{v_{k}^{\prime }}\right), \end{array} \end{array}$$

where

(A3) $$\begin{array}{c} \begin{array}{cc} \frac{\partial \Delta_{k}\left(\cdot \right)}{\partial u_{k}^{\prime }} & =\sum_{m=1}^{M}\{\frac{1}{m}\prod_{i=1}^{m}\frac{\tau_{k_{1}}+i-1}{\tau_{k_{1}}+\tau_{k_{2}}+i-1}\\ & \times {\left(\frac{v_{k}^{\prime }}{v_{k}^{\prime }+m}\right)}^{u_{k}^{\prime }}\left(\ln\left(v_{k}^{\prime }\right)-\ln\left(v_{k}^{\prime }+m\right)\right)\},\\ \frac{\partial \Delta_{k}\left(\cdot \right)}{\partial v_{k}^{\prime }} & =\sum_{m=1}^{M}\{\frac{1}{m}\prod_{i=1}^{m}\frac{\tau_{k_{1}}+i-1}{\tau_{k_{1}}+\tau_{k_{2}}+i-1}\\ & \times u_{k}^{\prime }{\left(\frac{v_{k}^{\prime }}{v_{k}^{\prime }+m}\right)}^{u_{k}^{\prime }-1}\frac{m}{{\left({v_{k}^{\prime }}^{2}+m\right)}^{2}}. \end{array} \end{array}$$

### Appendix C.5. Coordinate Update for q(α)

The update formulae for $\left(k_{1},k_{2}\right)$ are

(A4) $$\begin{array}{c} \begin{array}{cc} & k_{1}=K+\sum_{k=1}^{K}\sum_{r>1}^{R}\left(r-1\right)\varphi_{k}\left(r\right)+a_{1},\\ & k_{2}=-\sum_{k=1}^{K}E\left[\ln\left(1-V_{k}\right)\right]+\sum_{k=1}^{K}E\left[T_{k}\right]\varphi_{k}\left(r>1\right)+a_{2}. \end{array} \end{array}$$

It is shown that $\varphi_{k}\left(1\right)$ has nothing to do with the update of α.

### Appendix C.6. Coordinate Update for q(γ)

The update formulae for $\left(\tau_{1},\tau_{2}\right)$ are

(A5) $$\begin{array}{c} \begin{array}{cc} & \tau_{1}=K+b_{1},\\ & \tau_{2}=\sum_{r=1}^{R}\left\{1-\prod_{k=1}^{K}\sum_{r^{\prime }=1}^{r-1}\varphi_{k}\left(r^{\prime }\right)\right\}+b_{2}. \end{array} \end{array}$$

It can be seen from above that $\tau_{1}$ does not change with iterations while $\tau_{2}$ depends on $\varphi_{k}\left(r^{\prime }\right)$.

### Appendix C.7. Coordinate Update for q(**μ**_k_,*Σ*_k_)

The variational parameter update of $q\left(\mu_{k},\Sigma_{k}\right)$ is analytical and the update formulae are

$$\begin{array}{c} \begin{array}{cc} u_{k}= & \frac{\sum_{n}^{N}\sum_{t}^{T}q\left(z_{t}^{\left(n\right)}=k\right)x_{t}^{\left(n\right)}+\lambda_{0}u_{0}}{\lambda_{0}+\sum_{n}^{N}\sum_{t}^{T}q\left(z_{t}^{\left(n\right)}=k\right)},\\ \lambda_{k}= & \lambda_{0}+\sum_{n=1}^{N}\sum_{t=1}^{T}q\left(z_{t}^{\left(n\right)}=k\right),\\ \upsilon_{k}= & \upsilon_{0}+\sum_{n=1}^{N}\sum_{t=1}^{T}q\left(z_{t}^{\left(n\right)}=k\right),\\ \Phi_{k}= & \lambda_{0}u_{0}^{⊤}u_{0}+\sum_{n=1}^{N}\sum_{t=1}^{T}q\left(z_{t}^{\left(n\right)}=k\right)x_{t}^{{\left(n\right)}^{⊤}}x_{t}^{\left(n\right)}\\ & +\Phi_{0}-\frac{1}{\lambda_{0}+\sum_{n}^{N}\sum_{t}^{T}q\left(z_{t}^{\left(n\right)}=k\right)}\\ & \times {\left(\sum_{n}^{N}\sum_{t}^{T}q\left(z_{t}^{\left(n\right)}=k\right)x_{t}^{\left(n\right)}+\lambda_{0}u_{0}\right)}^{⊤}\\ & \times \left(\sum_{n}^{N}\sum_{t}^{T}q\left(z_{t}^{\left(n\right)}=k\right)x_{t}^{\left(n\right)}+\lambda_{0}u_{0}\right). \end{array} \end{array}$$

### Appendix C.8. Coordinate Update for q(**π**_j_^(c)^)

In order to update $\pi_{j}^{\left(c\right)}$, two cases, j>0 and j=0, should be analyzed. For j>0, the logarithmic distribution of $\pi_{j}^{\left(c\right)}$ is updated as

$$\begin{array}{c} \begin{array}{cc} \ln q\left(\pi_{j}^{\left(c\right)}\right) & =E_{q}[\ln\left(p\left(\pi_{j}|f_{n},r,\kappa \right)\right.\\ & \times \prod_{n=1}^{N}\prod_{t=1}^{T-1}\delta \left(y_{n}=c\right)P\left(z_{t+1}^{\left(n\right)}|\pi ,z_{t}^{\left(n\right)}\right)], \end{array} \end{array}$$

where

$$p\left(z_{t+1}^{\left(n\right)}|\pi_{k}^{\left(y_{n}\right)},z_{t}^{\left(n\right)}=j\right)=\prod_{k=1}^{K}{\pi_{j_{k}}^{\left(y_{n}\right)}}^{\delta \left(z_{t}^{\left(n\right)}=j,z_{t+1}^{\left(n\right)}=k\right)},$$

and

$$p\left(\pi_{j}^{\left(c\right)}|f_{ck},r^{\left(c\right)},\kappa \right)=\frac{1}{B}\prod_{k\neq j}^{K}{\pi_{jk}^{\left(c\right)}}^{\left(r^{\left(c\right)}f_{ck}-1\right)}{\pi_{jj}^{\left(c\right)}}^{\left(\left(r^{\left(c\right)}+\kappa \right)f_{ck}-1\right)}.$$

Here *B* is the normalizing constant of the Dirichlet distribution $q\left(\pi_{j}^{\left(c\right)}\right)$. We can get

$$\begin{array}{c} \begin{array}{c} \ln q\left(\pi_{j}^{\left(c\right)}\right)=\sum_{k\neq j}^{K}\{\sum_{\left(n=1\right)}^{N}\sum_{t=1}^{T-1}\delta \left(y_{n}=c\right)q\left(z_{t}^{\left(n\right)}=j,z_{t+1}^{\left(n\right)}=k\right)\\ \;+r\upsilon_{ck}-1\}\ln\left(\pi_{jk}^{\left(c\right)}\right)+(\left(r+\kappa \right)\upsilon_{ck}-1\\ \;+\sum_{n=1}^{N}\sum_{t}^{T-1}\delta \left(y_{n}=c\right)q\left(z_{t}^{\left(n\right)}=j,z_{t+1}^{\left(n\right)}=j\right))\ln\left(\pi_{jj}^{\left(c\right)}\right). \end{array} \end{array}$$

Thus the parameters $r_{j}^{\prime (c)}$ for the Dirichlet distribution $q\left(\pi_{j}^{\left(c\right)}\right)$ are formulated as

$$\begin{array}{c} \begin{array}{cc} r_{jk}^{\prime (c)}= & \sum_{n=1}^{N}\sum_{t=1}^{T-1}\delta \left(y_{n}=c\right)q\left(z_{t}^{\left(n\right)}=j,z_{t+1}^{\left(n\right)}=k\right)+r^{\left(c\right)}\upsilon_{ck},\\ & \mathrm{with}\;k\neq j, \end{array} \end{array}$$

and

$$\begin{array}{c} \begin{array}{cc} r_{jj}^{\prime (c)}= & \sum_{n=1}^{N}\sum_{t=1}^{T-1}\delta \left(y_{n}=c\right)q\left(z_{t}^{\left(n\right)}=j,z_{t+1}^{\left(n\right)}=j\right)+\left(r^{\left(c\right)}+\kappa \right)\upsilon_{cj}. \end{array} \end{array}$$

For j=0, $\pi_{j}^{\left(c\right)}$ is the prior probability of the hidden states. We can obtain

$$r_{0k}=\sum_{n=1}^{N}\delta \left(y_{n}=c\right)q\left(z_{1}^{\left(n\right)}=k\right)+r^{\left(c\right)}\upsilon_{ck}.$$

### Appendix C.9. Coordinate Update for q(Z)

For each class of trajectories,

$$\begin{array}{c} \begin{array}{cc} {a_{jk}^{*}}^{\left(c\right)} & =\exp\left(E_{q}\ln p\left(\pi_{jk}^{\left(c\right)}\right)\right)\\ & =\exp\left(\Psi \left(r_{jk}^{\prime (c)}\right)-\Psi \left(\sum_{i=1}^{K}r_{ji}^{\prime (c)}\right)\right),\\ {b_{tj}^{*}}^{\left(n\right)} & =\exp\left(E_{q}\ln p\left(x_{t}^{\left(n\right)}|\mu_{j},\Sigma_{j}\right)\right)\\ & =\exp\{-\frac{p}{2}\ln\left(2\pi \right)-\frac{1}{2}E_{q}\ln\left(|\Sigma_{j}|\right)\\ & -\frac{1}{2}\left(\frac{d}{\lambda_{k}}+{\left(x_{t}^{\left(n\right)}-u_{j}\right)}^{⊤}\left(\upsilon_{j}-p-1\right)\Phi_{j}^{-1}\left(x_{t}^{\left(n\right)}-u_{j}\right)\right)\}, \end{array} \end{array}$$

where *p* is the dimension of $x_{t}^{\left(n\right)}$ and

$$E_{q}\ln\left(|\Sigma_{j}|\right)=-\sum_{i=1}^{p}\Psi \left(\frac{\upsilon_{j}+1-i}{2}\right)-p\ln\left(2\right)+\ln\left|\Phi_{j}\right|.$$

From the above, we need the marginal probabilities $q\left(z_{t}^{\left(n\right)}=j\right)$ and $q\left(z_{t}^{\left(n\right)}=j,z_{t+1}^{\left(n\right)}=k\right)$. The detailed calculations are as follows. Both of them can be calculated by the forward-backward algorithm. The forward procedure is

$$\begin{array}{c} \begin{array}{cc} & \iota_{k}^{t}=P\left(x_{1}=x_{1},x_{2}=x_{2},\cdots ,x_{t}=x_{t},z_{t}k|W,\Theta \right),\\ & \iota_{k}^{1}=a_{0j}^{*}b_{tk}^{*},\\ & \iota_{j}^{t+1}=b_{t+1j}^{*}\sum_{k=1}^{K}\iota_{k}^{t}a_{kj}^{*}. \end{array} \end{array}$$

The backward procedure is

$$\begin{array}{c} \begin{array}{cc} & \beta_{k}\left(t\right)=P\left(x_{t+1}=x_{t+1},\cdots ,x_{T}=x_{T}|z_{t}=k,W,\Theta \right),\\ & \beta_{k}\left(T\right)=1,\\ & \beta_{k}\left(T\right)=\sum_{j=1}^{K}\beta_{j}\left(t+1\right)a_{kj}^{*}b_{t+1j}^{*}. \end{array} \end{array}$$

Thus, the expressions of the posterior distributions are

$$\begin{array}{c} \begin{array}{cc} & q\left(z_{t}=j\right)=\frac{\iota_{j}\left(t\right)\beta_{j}\left(t\right)}{\sum_{j=1}^{K}\iota_{j}\left(t\right)\beta_{j}\left(t\right)},\\ & q\left(z_{t}=j,z_{t+1}=k\right)=\frac{\iota_{j}\left(t\right)a_{jk}^{*}\beta_{j}\left(t\right)b_{t+1k}^{*}}{\sum_{k=1}^{K}\iota_{k}\left(t\right)\beta_{k}\left(t\right)}. \end{array} \end{array}$$

Now we can update the variational parameters in the BP-HMM according to the above equations. We judge the convergence of this update according to the change of the lower bound.
